# *r*-2,*c*-6-Bis(4-methoxy­phen­yl)-*c*-3,*t*-3-dimethyl­piperidin-4-one

**DOI:** 10.1107/S1600536808036830

**Published:** 2008-11-13

**Authors:** S. Ponnuswamy, V. Mohanraj, P. Gayathri, A. Thiruvalluvar, R. J. Butcher

**Affiliations:** a Department of Chemistry, Government Arts College (Autonomous), Coimbatore 641 018, Tamilnadu, India; b PG Research Department of Physics, Rajah Serfoji Government College (Autonomous), Thanjavur 613 005, Tamilnadu, India; c Department of Chemistry, Howard University, 525 College Street NW, Washington, DC 20059, USA

## Abstract

The asymmetric unit of the title compound, C_21_H_25_NO_3_, contains two crystallographically independent mol­ecules (*A* and *B*). In both mol­ecules, the piperidine ring adopts a chair conformation, with the methoxy­phenyl rings attached equatorially. The dihedral angle between the two benzene rings in mol­ecule *A* is 73.79 (8)°; the corresponding value in mol­ecule *B* is 77.71 (8)°. The mol­ecules are linked by N—H⋯O hydrogen bonds. In addition, C—H⋯π inter­actions are also found in the crystal structure.

## Related literature

For a related crystal structure, see: Gayathri *et al.* (2008 ▶). For the biological and pharmacological activities of piperidones, see: Dimmock *et al.* (1990 ▶); Mutus *et al.* (1989 ▶).
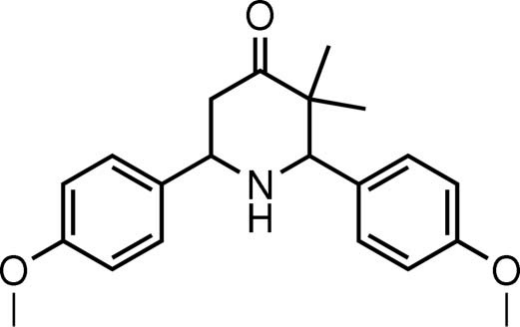


## Experimental

### 

#### Crystal data

C_21_H_25_NO_3_
*M* *_r_* = 339.42
Monoclinic, 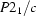

*a* = 5.9772 (2) Å
*b* = 23.0858 (8) Å
*c* = 26.7611 (8) Å
β = 93.543 (3)°
*V* = 3685.7 (2) Å^3^
*Z* = 8
Mo *K*α radiation
μ = 0.08 mm^−1^
*T* = 200 (2) K
0.47 × 0.39 × 0.21 mm

#### Data collection

Oxford Diffraction Gemini R diffractometer
Absorption correction: multi-scan (*CrysAlis RED*; Oxford Diffraction, 2008 ▶) *T*_min_ = 0.866, *T*_max_ = 1.000 (expected range = 0.852–0.983)
31849 measured reflections
12080 independent reflections
3437 reflections with *I* > 2σ(*I*)
*R*_int_ = 0.087

#### Refinement

*R*[*F* ^2^ > 2σ(*F* ^2^)] = 0.044
*wR*(*F* ^2^) = 0.100
*S* = 0.74
12080 reflections
459 parameters
H atoms treated by a mixture of independent and constrained refinement
Δρ_max_ = 0.19 e Å^−3^
Δρ_min_ = −0.19 e Å^−3^

### 

Data collection: *CrysAlis CCD* (Oxford Diffraction, 2008 ▶); cell refinement: *CrysAlis RED* (Oxford Diffraction, 2008 ▶); data reduction: *CrysAlis RED*; program(s) used to solve structure: *SHELXS97* (Sheldrick, 2008 ▶); program(s) used to refine structure: *SHELXL97* (Sheldrick, 2008 ▶); molecular graphics: *ORTEP-3* (Farrugia, 1997 ▶); software used to prepare material for publication: *PLATON* (Spek, 2003 ▶).

## Supplementary Material

Crystal structure: contains datablocks global, I. DOI: 10.1107/S1600536808036830/wn2288sup1.cif

Structure factors: contains datablocks I. DOI: 10.1107/S1600536808036830/wn2288Isup2.hkl

Additional supplementary materials:  crystallographic information; 3D view; checkCIF report

## Figures and Tables

**Table 1:** Hydrogen-bond geometry (Å, °)

*D*—H⋯*A*	*D*—H	H⋯*A*	*D*⋯*A*	*D*—H⋯*A*
N1*A*—H1*A*⋯O4*B*	0.92 (2)	2.28 (2)	3.1958 (17)	173.2 (14)
C25*B*—H25*B*⋯*Cg*1^i^	0.95	2.95	3.6993 (19)	137
C32*A*—H32*B*⋯*Cg*2^ii^	0.98	2.82	3.4573 (19)	124
C5*B*—H52*B*⋯*Cg*1	0.99	2.97	3.7989 (19)	142

Symmetry codes: (i) 
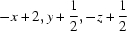
; (ii) 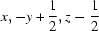. *Cg*1 and *Cg*2 are the centroids of the C61*A*–C66*A* and C61*B*–C66*B* rings, respectively.

**Fig. 1.**
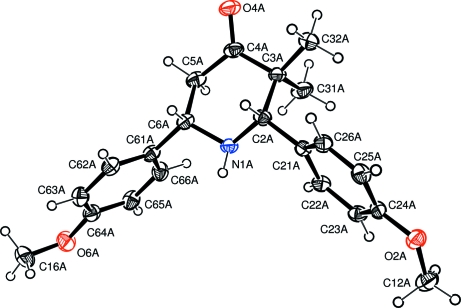
The molecular structure of the independent molecule A, showing the atom-numbering scheme and displacement ellipsoids drawn at the 30% probability level (arbitrary spheres for H atoms).

**Fig. 2.**
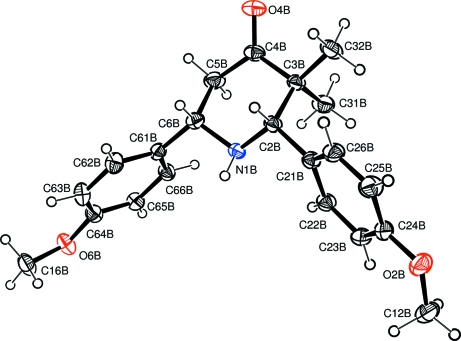
The molecular structure of the independent molecule B, showing the atom-numbering scheme and displacement ellipsoids drawn at the 30% probability level (arbitrary spheres for H atoms).

**Fig. 3.**
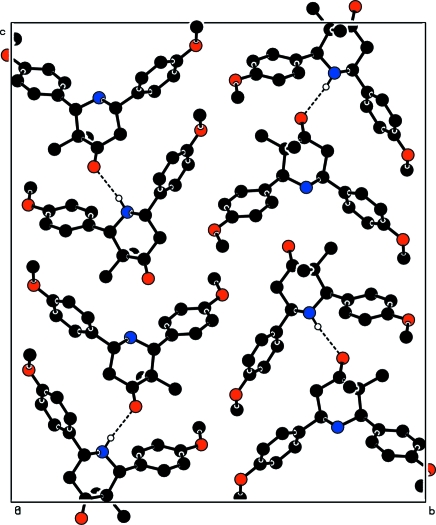
The packing of the title compound, viewed down the a axis. Dashed lines indicate hydrogen bonds. H atoms not involved in hydrogen bonding have been omitted.

## supplementary crystallographic information

### Comment

2,6-Disubstituted 4-piperidones have various biological and pharmacological activities (Dimmock *et al.*, 1990; Mutus *et al.*, 1989). The crystal structure of r-2,c-6-bis(4-chlorophenyl)-t-3-isopropyl-1-nitrosopiperidin-4-one has been reported, in which the piperidine ring adopts a chair conformation (Gayathri *et al.*, 2008).

The asymmetric unit of the title compound, C_21_H_25_NO_3_, contains two crystallographically independent molecules A and B. In both molecules, the piperidine ring adopts a chair conformation, with the methoxyphenyl rings and one of the methyl groups attached equatorially. The dihedral angle between the two benzene rings in molecule A is 73.79 (8)°; the corresponding value in molecule B is 77.71 (8)°. Both molecules are nearly identical, the r.m.s deviation of an overlay of all non-hydrogen atoms being 0.127 Å. The molecules are linked by N1A—H1A···O4B hydrogen bonds. Furthermore, C25B—H25B···π, C32A—H32B···π and C5B—H52B···π interactions are also found in the crystal structure (Fig. 3, Table 1).

### Experimental

Anisaldehyde (24.2 ml, 0.20 mol), 3-methyl-2-butanone (10.7 ml, 0.10 mol) and ammonium acetate (7.7 g, 0.10 mol) were dissolved in 80 ml of distilled ethanol and heated over a boiling water bath, with shaking until a yellow colour developed, which changed to orange. The solution was left undisturbed for 14 h. The precipitated solid was filtered and purified by recrystallization from ethanol. The yield obtained was 72.4% (24.6 g).

### Refinement

Atoms H1A at N1A and H1B at N1B were located in a difference Fourier map and refined isotropically; N1A—H1A = 0.92 (2) Å and N1B—H1B = 0.87 (2) Å. The remaining H atoms were positioned geometrically and allowed to ride on their parent atoms, with C—H = 0.95, 0.98, 0.99 and 1.00 Å for C*sp*^2^, methyl, methylene and methine C, respectively; *U*_iso_(H) = k*U*_eq_(C), where k = 1.5 for methyl and 1.2 for all other H atoms.

### Crystal data

**Table:**

C_21_H_25_NO_3_	*F*_000_ = 1456
*M**_r_* = 339.42	*D*_x_ = 1.223 Mg m^−^^3^
Monoclinic, *P*2_1_/*c*	Melting point: 344(1) K
Hall symbol: -P 2ybc	Mo *K*α radiation λ = 0.71073 Å
*a* = 5.9772 (2) Å	Cell parameters from 4399 reflections
*b* = 23.0858 (8) Å	θ = 4.6–32.5º
*c* = 26.7611 (8) Å	µ = 0.08 mm^−^^1^
β = 93.543 (3)º	*T* = 200 (2) K
*V* = 3685.7 (2) Å^3^	Rectangular prism, colourless
*Z* = 8	0.47 × 0.39 × 0.21 mm

### Data collection

**Table:**

Oxford Diffraction R Gemini diffractometer	12080 independent reflections
Radiation source: fine-focus sealed tube	3437 reflections with *I* > 2σ(*I*)
Monochromator: graphite	*R*_int_ = 0.087
Detector resolution: 10.5081 pixels mm^-1^	θ_max_ = 32.6º
*T* = 200(2) K	θ_min_ = 4.6º
φ and ω scans	*h* = −8→8
Absorption correction: multi-scan(CrysAlis RED; Oxford Diffraction, 2008)	*k* = −34→34
*T*_min_ = 0.866, *T*_max_ = 1.000	*l* = −40→36
31849 measured reflections	

### Refinement

**Table:**

Refinement on *F*^2^	Secondary atom site location: difference Fourier map
Least-squares matrix: full	Hydrogen site location: inferred from neighbouring sites
*R*[*F*^2^ > 2σ(*F*^2^)] = 0.045	H atoms treated by a mixture of independent and constrained refinement
*wR*(*F*^2^) = 0.100	*w* = 1/[σ^2^(*F*_o_^2^) + (0.0352*P*)^2^] where *P* = (*F*_o_^2^ + 2*F*_c_^2^)/3
*S* = 0.74	(Δ/σ)_max_ = 0.001
12080 reflections	Δρ_max_ = 0.19 e Å^−^^3^
459 parameters	Δρ_min_ = −0.19 e Å^−^^3^
Primary atom site location: structure-invariant direct methods	Extinction correction: none

### Special details

**Table:**

Geometry. Bond distances, angles *etc*. have been calculated using the rounded fractional coordinates. All su's are estimated from the variances of the (full) variance-covariance matrix. The cell e.s.d.'s are taken into account in the estimation of distances, angles and torsion angles
Refinement. Refinement of *F*^2^ against ALL reflections. The weighted *R*-factor *wR* and goodness of fit *S* are based on *F*^2^, conventional *R*-factors *R* are based on *F*, with *F* set to zero for negative *F*^2^. The threshold expression of *F*^2^ > 2σ(*F*^2^) is used only for calculating *R*-factors(gt) *etc*. and is not relevant to the choice of reflections for refinement. *R*-factors based on *F*^2^ are statistically about twice as large as those based on *F*, and *R*- factors based on ALL data will be even larger.

### Fractional atomic coordinates and isotropic or equivalent isotropic displacement parameters (Å^2^)

**Table:**

	*x*	*y*	*z*	*U*_iso_*/*U*_eq_	
O2A	0.34797 (17)	0.45889 (5)	0.11995 (4)	0.0454 (4)	
O4A	0.99167 (18)	0.16935 (5)	−0.03347 (4)	0.0541 (5)	
O6A	1.13775 (19)	0.04235 (5)	0.27582 (4)	0.0563 (5)	
N1A	0.8561 (2)	0.21976 (6)	0.10498 (5)	0.0343 (5)	
C2A	0.8410 (2)	0.26156 (7)	0.06365 (5)	0.0331 (5)	
C3A	0.7590 (2)	0.23032 (7)	0.01421 (5)	0.0364 (6)	
C4A	0.9137 (3)	0.17926 (8)	0.00670 (6)	0.0410 (6)	
C5A	0.9674 (3)	0.14121 (7)	0.05153 (6)	0.0449 (6)	
C6A	1.0314 (3)	0.17631 (7)	0.09858 (6)	0.0355 (6)	
C12A	0.1806 (3)	0.45333 (8)	0.15555 (6)	0.0533 (7)	
C16A	1.3376 (3)	0.04489 (9)	0.30714 (7)	0.0666 (8)	
C21A	0.7009 (2)	0.31252 (7)	0.07778 (5)	0.0313 (6)	
C22A	0.5117 (3)	0.30645 (7)	0.10449 (5)	0.0358 (6)	
C23A	0.3878 (2)	0.35387 (7)	0.11913 (5)	0.0348 (6)	
C24A	0.4556 (3)	0.40885 (7)	0.10728 (5)	0.0343 (6)	
C25A	0.6440 (3)	0.41628 (8)	0.08030 (6)	0.0407 (6)	
C26A	0.7644 (3)	0.36880 (7)	0.06602 (5)	0.0376 (6)	
C31A	0.5213 (3)	0.20584 (8)	0.01718 (6)	0.0537 (7)	
C32A	0.7646 (3)	0.27174 (8)	−0.02963 (6)	0.0553 (7)	
C61A	1.0661 (3)	0.14007 (7)	0.14514 (6)	0.0366 (6)	
C62A	1.2642 (3)	0.14233 (7)	0.17425 (6)	0.0400 (6)	
C63A	1.2967 (3)	0.11037 (7)	0.21842 (6)	0.0442 (6)	
C64A	1.1263 (3)	0.07565 (7)	0.23337 (6)	0.0434 (6)	
C65A	0.9261 (3)	0.07281 (8)	0.20450 (7)	0.0471 (7)	
C66A	0.8967 (3)	0.10444 (8)	0.16116 (6)	0.0443 (7)	
O2B	0.3867 (2)	0.50938 (5)	0.43235 (4)	0.0595 (5)	
O4B	0.98221 (18)	0.30041 (5)	0.19962 (4)	0.0512 (5)	
O6B	1.06971 (18)	0.05432 (5)	0.45240 (4)	0.0526 (4)	
N1B	0.8531 (2)	0.28789 (6)	0.34345 (5)	0.0341 (5)	
C2B	0.8492 (2)	0.34464 (7)	0.31903 (5)	0.0326 (5)	
C3B	0.7574 (2)	0.33823 (7)	0.26337 (5)	0.0358 (6)	
C4B	0.8988 (3)	0.29235 (8)	0.23961 (6)	0.0398 (6)	
C5B	0.9335 (3)	0.23664 (8)	0.26753 (6)	0.0480 (7)	
C6B	1.0127 (3)	0.24800 (7)	0.32211 (6)	0.0373 (6)	
C12B	0.2267 (3)	0.49004 (9)	0.46597 (7)	0.0654 (8)	
C16B	1.2452 (3)	0.05251 (9)	0.49107 (6)	0.0583 (7)	
C21B	0.7197 (2)	0.38739 (7)	0.34875 (5)	0.0341 (5)	
C22B	0.5352 (3)	0.37097 (7)	0.37446 (6)	0.0395 (6)	
C23B	0.4186 (3)	0.40994 (8)	0.40256 (6)	0.0428 (6)	
C24B	0.4860 (3)	0.46684 (8)	0.40507 (6)	0.0454 (7)	
C25B	0.6680 (3)	0.48488 (8)	0.37891 (6)	0.0516 (7)	
C26B	0.7830 (3)	0.44501 (8)	0.35184 (6)	0.0447 (6)	
C31B	0.5139 (3)	0.31671 (8)	0.25968 (6)	0.0513 (7)	
C32B	0.7731 (3)	0.39571 (8)	0.23597 (6)	0.0513 (7)	
C61B	1.0357 (3)	0.19431 (7)	0.35393 (6)	0.0351 (6)	
C62B	1.2267 (3)	0.18511 (7)	0.38464 (6)	0.0436 (6)	
C63B	1.2468 (3)	0.13897 (8)	0.41753 (6)	0.0467 (7)	
C64B	1.0700 (3)	0.10078 (7)	0.42025 (6)	0.0399 (6)	
C65B	0.8794 (3)	0.10823 (7)	0.38913 (6)	0.0398 (6)	
C66B	0.8624 (3)	0.15445 (7)	0.35672 (6)	0.0394 (6)	
H1A	0.893 (2)	0.2402 (7)	0.1339 (6)	0.051 (5)*	
H2A	0.99594	0.27622	0.05916	0.0398*	
H6A	1.17461	0.19717	0.09323	0.0426*	
H12A	0.11441	0.49137	0.16147	0.0799*	
H12B	0.06341	0.42668	0.14251	0.0799*	
H12C	0.24914	0.43812	0.18708	0.0799*	
H16A	1.32401	0.01930	0.33602	0.0998*	
H16B	1.46443	0.03237	0.28827	0.0998*	
H16C	1.36262	0.08474	0.31880	0.0998*	
H22A	0.46465	0.26865	0.11316	0.0429*	
H23A	0.25745	0.34831	0.13715	0.0417*	
H25A	0.69029	0.45415	0.07164	0.0488*	
H26A	0.89383	0.37454	0.04772	0.0451*	
H31A	0.51819	0.17917	0.04562	0.0806*	
H31B	0.41603	0.23770	0.02166	0.0806*	
H31C	0.47798	0.18504	−0.01385	0.0806*	
H32A	0.66412	0.30445	−0.02444	0.0829*	
H32B	0.91775	0.28607	−0.03224	0.0829*	
H32C	0.71546	0.25149	−0.06057	0.0829*	
H51A	0.83532	0.11688	0.05758	0.0539*	
H52A	1.09280	0.11509	0.04439	0.0539*	
H62A	1.38203	0.16632	0.16399	0.0480*	
H63A	1.43481	0.11261	0.23789	0.0530*	
H65A	0.80829	0.04879	0.21477	0.0565*	
H66A	0.75844	0.10198	0.14176	0.0532*	
H1B	0.896 (2)	0.2920 (7)	0.3749 (6)	0.043 (5)*	
H2B	1.00732	0.35876	0.31891	0.0391*	
H6B	1.16216	0.26753	0.32261	0.0448*	
H12D	0.16706	0.52341	0.48346	0.0981*	
H12E	0.10386	0.47004	0.44712	0.0981*	
H12F	0.29901	0.46335	0.49044	0.0981*	
H16D	1.22896	0.01764	0.51142	0.0874*	
H16E	1.39058	0.05170	0.47604	0.0874*	
H16F	1.23651	0.08693	0.51229	0.0874*	
H22B	0.48725	0.33175	0.37277	0.0475*	
H23B	0.29334	0.39741	0.41990	0.0513*	
H25B	0.71265	0.52437	0.37970	0.0619*	
H26B	0.90925	0.45750	0.33482	0.0536*	
H31D	0.41757	0.34543	0.27466	0.0769*	
H31E	0.46438	0.31110	0.22441	0.0769*	
H31F	0.50427	0.27986	0.27760	0.0769*	
H32D	0.67997	0.42461	0.25166	0.0770*	
H32E	0.92932	0.40885	0.23775	0.0770*	
H32F	0.72019	0.39070	0.20084	0.0770*	
H51B	0.79127	0.21458	0.26633	0.0575*	
H52B	1.04663	0.21296	0.25133	0.0575*	
H62B	1.34833	0.21140	0.38311	0.0522*	
H63B	1.38066	0.13359	0.43803	0.0560*	
H65B	0.75950	0.08133	0.39011	0.0477*	
H66B	0.72948	0.15926	0.33579	0.0473*	

### Atomic displacement parameters (Å^2^)

**Table:**

	*U*^11^	*U*^22^	*U*^33^	*U*^12^	*U*^13^	*U*^23^
O2A	0.0505 (7)	0.0399 (8)	0.0464 (7)	0.0074 (6)	0.0077 (6)	−0.0011 (6)
O4A	0.0528 (7)	0.0718 (10)	0.0386 (7)	0.0077 (7)	0.0099 (6)	−0.0160 (6)
O6A	0.0631 (8)	0.0521 (9)	0.0540 (8)	0.0058 (7)	0.0063 (7)	0.0173 (7)
N1A	0.0429 (8)	0.0325 (9)	0.0280 (8)	0.0028 (7)	0.0057 (7)	−0.0020 (7)
C2A	0.0319 (9)	0.0377 (10)	0.0301 (9)	−0.0046 (8)	0.0042 (7)	0.0011 (8)
C3A	0.0341 (9)	0.0478 (11)	0.0274 (9)	0.0013 (9)	0.0036 (7)	−0.0045 (8)
C4A	0.0359 (10)	0.0518 (12)	0.0357 (10)	−0.0056 (9)	0.0052 (8)	−0.0149 (9)
C5A	0.0512 (10)	0.0410 (11)	0.0435 (11)	0.0023 (9)	0.0108 (8)	−0.0082 (9)
C6A	0.0366 (9)	0.0319 (10)	0.0390 (10)	0.0018 (8)	0.0096 (7)	−0.0007 (8)
C12A	0.0455 (10)	0.0593 (13)	0.0559 (12)	0.0027 (10)	0.0099 (9)	−0.0118 (10)
C16A	0.0769 (14)	0.0720 (16)	0.0493 (12)	0.0099 (12)	−0.0078 (11)	0.0159 (11)
C21A	0.0308 (9)	0.0368 (11)	0.0261 (9)	0.0018 (8)	0.0011 (7)	0.0013 (8)
C22A	0.0413 (10)	0.0337 (10)	0.0328 (9)	−0.0060 (9)	0.0052 (8)	−0.0007 (8)
C23A	0.0331 (9)	0.0406 (11)	0.0309 (9)	−0.0058 (9)	0.0047 (7)	−0.0023 (8)
C24A	0.0365 (9)	0.0348 (11)	0.0311 (9)	0.0047 (9)	−0.0027 (7)	−0.0003 (8)
C25A	0.0449 (10)	0.0357 (11)	0.0417 (10)	−0.0040 (9)	0.0036 (8)	0.0112 (9)
C26A	0.0365 (9)	0.0417 (12)	0.0351 (10)	−0.0006 (9)	0.0073 (7)	0.0057 (9)
C31A	0.0378 (10)	0.0767 (15)	0.0470 (11)	−0.0055 (10)	0.0055 (8)	−0.0226 (10)
C32A	0.0658 (12)	0.0645 (14)	0.0359 (11)	0.0176 (10)	0.0057 (9)	0.0023 (10)
C61A	0.0388 (10)	0.0303 (10)	0.0417 (10)	0.0020 (9)	0.0103 (8)	−0.0007 (8)
C62A	0.0391 (10)	0.0324 (10)	0.0494 (11)	−0.0008 (9)	0.0093 (9)	−0.0017 (9)
C63A	0.0425 (10)	0.0406 (12)	0.0490 (11)	0.0031 (9)	−0.0003 (8)	0.0020 (9)
C64A	0.0510 (11)	0.0357 (11)	0.0443 (11)	0.0078 (10)	0.0098 (9)	0.0054 (9)
C65A	0.0398 (10)	0.0432 (12)	0.0596 (12)	−0.0008 (9)	0.0130 (9)	0.0093 (10)
C66A	0.0378 (10)	0.0432 (12)	0.0520 (12)	0.0002 (9)	0.0032 (8)	0.0066 (10)
O2B	0.0756 (9)	0.0433 (8)	0.0625 (9)	0.0166 (7)	0.0272 (7)	0.0037 (7)
O4B	0.0599 (8)	0.0637 (9)	0.0308 (7)	−0.0078 (7)	0.0104 (6)	−0.0002 (6)
O6B	0.0638 (8)	0.0435 (8)	0.0486 (7)	0.0014 (7)	−0.0118 (6)	0.0089 (6)
N1B	0.0410 (8)	0.0355 (9)	0.0258 (8)	0.0039 (7)	0.0033 (7)	0.0037 (7)
C2B	0.0335 (9)	0.0342 (10)	0.0302 (9)	−0.0016 (8)	0.0032 (7)	0.0047 (8)
C3B	0.0356 (9)	0.0414 (11)	0.0308 (9)	−0.0024 (8)	0.0045 (7)	0.0079 (8)
C4B	0.0388 (9)	0.0510 (12)	0.0295 (10)	−0.0102 (9)	0.0012 (8)	−0.0014 (9)
C5B	0.0592 (11)	0.0495 (13)	0.0361 (10)	0.0016 (10)	0.0106 (8)	−0.0046 (9)
C6B	0.0345 (9)	0.0407 (11)	0.0372 (10)	−0.0012 (8)	0.0066 (8)	−0.0001 (9)
C12B	0.0748 (13)	0.0640 (14)	0.0608 (13)	0.0176 (12)	0.0316 (11)	0.0027 (11)
C16B	0.0679 (12)	0.0560 (13)	0.0488 (12)	0.0137 (10)	−0.0139 (10)	0.0079 (10)
C21B	0.0351 (9)	0.0332 (10)	0.0341 (9)	−0.0019 (8)	0.0042 (7)	0.0053 (8)
C22B	0.0428 (10)	0.0374 (11)	0.0390 (10)	−0.0033 (9)	0.0071 (8)	0.0048 (9)
C23B	0.0429 (10)	0.0467 (12)	0.0401 (10)	0.0002 (10)	0.0129 (8)	0.0087 (9)
C24B	0.0538 (11)	0.0405 (12)	0.0431 (11)	0.0133 (10)	0.0125 (9)	0.0072 (9)
C25B	0.0663 (12)	0.0361 (11)	0.0538 (12)	−0.0023 (10)	0.0157 (10)	0.0033 (10)
C26B	0.0474 (10)	0.0420 (12)	0.0463 (11)	−0.0023 (10)	0.0163 (8)	0.0080 (9)
C31B	0.0437 (10)	0.0721 (14)	0.0381 (10)	−0.0084 (10)	0.0017 (8)	0.0035 (9)
C32B	0.0655 (12)	0.0513 (13)	0.0372 (10)	0.0009 (10)	0.0042 (9)	0.0116 (9)
C61B	0.0342 (9)	0.0343 (10)	0.0374 (10)	0.0049 (9)	0.0064 (8)	−0.0009 (8)
C62B	0.0350 (10)	0.0400 (11)	0.0555 (11)	−0.0034 (9)	0.0016 (9)	−0.0010 (10)
C63B	0.0400 (10)	0.0417 (12)	0.0568 (12)	0.0061 (10)	−0.0089 (9)	0.0019 (10)
C64B	0.0468 (11)	0.0349 (11)	0.0376 (10)	0.0028 (10)	0.0004 (8)	−0.0013 (9)
C65B	0.0380 (10)	0.0391 (11)	0.0420 (10)	−0.0048 (9)	0.0003 (8)	−0.0010 (9)
C66B	0.0361 (9)	0.0440 (12)	0.0376 (10)	−0.0015 (9)	−0.0024 (8)	0.0033 (9)

### Geometric parameters (Å, °)

**Table:**

O2A—C12A	1.429 (2)	C31A—H31B	0.9800
O2A—C24A	1.375 (2)	C32A—H32B	0.9800
O4A—C4A	1.220 (2)	C32A—H32C	0.9800
O6A—C16A	1.417 (2)	C32A—H32A	0.9800
O6A—C64A	1.3699 (19)	C62A—H62A	0.9500
O2B—C12B	1.425 (2)	C63A—H63A	0.9500
O2B—C24B	1.380 (2)	C65A—H65A	0.9500
O4B—C4B	1.223 (2)	C66A—H66A	0.9500
O6B—C16B	1.428 (2)	C2B—C3B	1.5623 (19)
O6B—C64B	1.375 (2)	C2B—C21B	1.511 (2)
N1A—C2A	1.466 (2)	C3B—C31B	1.535 (2)
N1A—C6A	1.468 (2)	C3B—C32B	1.522 (2)
N1A—H1A	0.922 (16)	C3B—C4B	1.519 (2)
N1B—C6B	1.467 (2)	C4B—C5B	1.495 (3)
N1B—C2B	1.464 (2)	C5B—C6B	1.530 (2)
N1B—H1B	0.870 (16)	C6B—C61B	1.505 (2)
C2A—C3A	1.559 (2)	C21B—C22B	1.388 (2)
C2A—C21A	1.506 (2)	C21B—C26B	1.384 (2)
C3A—C31A	1.536 (2)	C22B—C23B	1.388 (2)
C3A—C32A	1.516 (2)	C23B—C24B	1.374 (3)
C3A—C4A	1.519 (2)	C24B—C25B	1.393 (2)
C4A—C5A	1.505 (2)	C25B—C26B	1.381 (3)
C5A—C6A	1.526 (2)	C61B—C66B	1.391 (2)
C6A—C61A	1.505 (2)	C61B—C62B	1.381 (2)
C21A—C26A	1.395 (2)	C62B—C63B	1.382 (2)
C21A—C22A	1.382 (2)	C63B—C64B	1.382 (3)
C22A—C23A	1.392 (2)	C64B—C65B	1.380 (2)
C23A—C24A	1.375 (2)	C65B—C66B	1.375 (2)
C24A—C25A	1.385 (2)	C2B—H2B	1.0000
C25A—C26A	1.378 (2)	C5B—H51B	0.9900
C61A—C66A	1.393 (2)	C5B—H52B	0.9900
C61A—C62A	1.378 (2)	C6B—H6B	1.0000
C62A—C63A	1.397 (2)	C12B—H12D	0.9800
C63A—C64A	1.375 (2)	C12B—H12E	0.9800
C64A—C65A	1.385 (3)	C12B—H12F	0.9800
C65A—C66A	1.373 (3)	C16B—H16D	0.9800
C2A—H2A	1.0000	C16B—H16E	0.9800
C5A—H51A	0.9900	C16B—H16F	0.9800
C5A—H52A	0.9900	C22B—H22B	0.9500
C6A—H6A	1.0000	C23B—H23B	0.9500
C12A—H12A	0.9800	C25B—H25B	0.9500
C12A—H12C	0.9800	C26B—H26B	0.9500
C12A—H12B	0.9800	C31B—H31D	0.9800
C16A—H16B	0.9800	C31B—H31E	0.9800
C16A—H16A	0.9800	C31B—H31F	0.9800
C16A—H16C	0.9800	C32B—H32D	0.9800
C22A—H22A	0.9500	C32B—H32E	0.9800
C23A—H23A	0.9500	C32B—H32F	0.9800
C25A—H25A	0.9500	C62B—H62B	0.9500
C26A—H26A	0.9500	C63B—H63B	0.9500
C31A—H31C	0.9800	C65B—H65B	0.9500
C31A—H31A	0.9800	C66B—H66B	0.9500
			
O2A···C16A^i^	3.291 (2)	H12B···C25A^xiv^	2.9300
O4B···N1A	3.1958 (17)	H12B···C23A	2.6700
O6A···C12A^ii^	3.413 (2)	H12B···C26A^xiv^	2.9500
O2A···H65B^iii^	2.9100	H12B···H23A	2.1600
O2A···H16A^i^	2.6300	H12C···C23A	2.8200
O2B···H51A^iii^	2.8300	H12C···H23A	2.4700
O2B···H12F^iv^	2.7800	H12C···H32E^xiv^	2.5000
O4A···H23B^v^	2.7300	H12D···C5A^iii^	2.9700
O4A···H1B^vi^	2.639 (16)	H12D···H12D^xi^	2.4800
O4A···H32C	2.5900	H12D···H51A^iii^	2.4200
O4A···H32B	2.7300	H12D···C12B^xi^	2.8000
O4B···H32F	2.6100	H12D···H12E^xi^	2.5400
O4B···H32E	2.7300	H12E···H23B	2.1700
O4B···H1A	2.279 (16)	H12E···C23B	2.6800
O4B···H23A^vii^	2.6600	H12E···H12D^xi^	2.5400
O6A···H12A^ii^	2.6000	H12F···O2B^iv^	2.7800
O6B···H25A^viii^	2.8200	H12F···C23B	2.7900
O6B···H16D^ix^	2.6600	H12F···H23B	2.4200
N1A···O4B	3.1958 (17)	H16A···O2A^viii^	2.6300
N1A···H22A	2.6200	H16B···C63A	2.7400
N1A···H31A	2.6600	H16B···H63A	2.2900
N1B···H31F	2.6500	H16C···C62B	3.0500
N1B···H22B	2.5800	H16C···C63A	2.7600
N1B···H32C^x^	2.8900	H16C···C63B	3.0400
C12A···O6A^iii^	3.413 (2)	H16C···H63A	2.3200
C12B···C12B^xi^	3.389 (3)	H16D···O6B^ix^	2.6600
C16A···O2A^viii^	3.291 (2)	H16E···H63B	2.1500
C16B···C25A^xii^	3.347 (2)	H16E···C63B	2.6600
C16B···C24A^xii^	3.401 (2)	H16F···H26A^x^	2.4700
C22A···C31A	3.298 (2)	H16F···C63B	2.8100
C22B···C31B	3.312 (2)	H16F···H63B	2.4600
C24A···C16B^xiii^	3.401 (2)	H16F···C24A^xii^	2.7900
C25A···C16B^xiii^	3.347 (2)	H16F···C25A^xii^	2.9500
C26A···C32A	3.402 (2)	H22A···N1A	2.6200
C26B···C32B	3.300 (2)	H22A···C31A	2.9900
C31A···C22A	3.298 (2)	H22A···H6A^xiv^	2.4300
C31B···C22B	3.312 (2)	H22A···H31B	2.5500
C32A···C26A	3.402 (2)	H22B···C31B	3.0600
C32A···C66B^vi^	3.566 (2)	H22B···N1B	2.5800
C32B···C26B	3.300 (2)	H23A···C12A	2.5200
C66B···C32A^x^	3.566 (2)	H23A···O4B^xiv^	2.6600
C4B···H1A	3.073 (16)	H23A···H12B	2.1600
C5A···H31A	2.8200	H23A···H12C	2.4700
C5A···H12D^ii^	2.9700	H23B···C12B	2.5100
C5A···H66A	2.9300	H23B···O4A^xv^	2.7300
C5B···H31F	2.7800	H23B···H12E	2.1700
C5B···H66B	2.8800	H23B···H12F	2.4200
C12A···H23A	2.5200	H25A···O6B^i^	2.8200
C12A···H32E^xiv^	2.9300	H25A···C16B^i^	2.8600
C12B···H12D^xi^	2.8000	H25B···C61A^i^	3.0700
C12B···H51A^iii^	3.0100	H25B···C62A^i^	3.0900
C12B···H23B	2.5100	H26A···H16F^vi^	2.4700
C16A···H63A	2.5200	H26A···H2A	2.3700
C16B···H63B	2.5100	H26B···C65A^i^	3.0500
C16B···H25A^viii^	2.8600	H26B···C32B	3.0700
C21A···H32A	2.7400	H26B···H2B	2.4000
C21A···H31B	2.8000	H31A···C5A	2.8200
C21B···H32D	2.7300	H31A···H6A^xiv^	2.5200
C21B···H31D	2.7700	H31A···H51A	2.3900
C22A···H1A	2.816 (14)	H31A···N1A	2.6600
C22A···H31B	2.7600	H31B···H22A	2.5500
C22B···H1B	2.823 (14)	H31B···H32A	2.5100
C22B···H31D	2.7800	H31B···C21A	2.8000
C23A···H32F	2.9900	H31B···C22A	2.7600
C23A···H12C	2.8200	H31C···H32C	2.4800
C23A···H31E	2.9900	H31D···C21B	2.7700
C23A···H12B	2.6700	H31D···H32D	2.5100
C23B···H12F	2.7900	H31D···C22B	2.7800
C23B···H12E	2.6800	H31E···H32F	2.5000
C24A···H16F^xiii^	2.7900	H31E···C23A	2.9900
C24A···H32F	2.9100	H31F···H51B	2.3200
C25A···H16F^xiii^	2.9500	H31F···N1B	2.6500
C25A···H12B^vii^	2.9300	H31F···C5B	2.7800
C26A···H12B^vii^	2.9500	H31F···H6B^xiv^	2.4500
C26A···H32A	2.8700	H32A···C26A	2.8700
C26B···H32D	2.7500	H32A···H31B	2.5100
C31A···H51A	2.9400	H32A···H63B^xiii^	2.3900
C31A···H6A^xiv^	3.0000	H32A···C21A	2.7400
C31A···H22A	2.9900	H32B···C64B^vi^	3.0700
C31B···H22B	3.0600	H32B···H2A	2.4700
C31B···H51B	2.8800	H32B···C62B^vi^	3.0500
C31B···H6B^xiv^	3.0000	H32B···O4A	2.7300
C32A···H1B^vi^	3.092 (16)	H32B···C63B^vi^	3.0000
C32B···H26B	3.0700	H32C···O4A	2.5900
C61A···H25B^viii^	3.0700	H32C···H31C	2.4800
C62A···H52B	2.9900	H32C···N1B^vi^	2.8900
C62A···H25B^viii^	3.0900	H32C···H1B^vi^	2.3200
C62B···H16C	3.0500	H32D···C21B	2.7300
C62B···H32B^x^	3.0500	H32D···C26B	2.7500
C63A···H52B	2.9600	H32D···H31D	2.5100
C63A···H16B	2.7400	H32E···O4B	2.7300
C63A···H16C	2.7600	H32E···H12C^vii^	2.5000
C63B···H16F	2.8100	H32E···C12A^vii^	2.9300
C63B···H16C	3.0400	H32E···H2B	2.4800
C63B···H16E	2.6600	H32F···O4B	2.6100
C63B···H32B^x^	3.0000	H32F···H31E	2.5000
C64B···H32B^x^	3.0700	H32F···C23A	2.9900
C65A···H26B^viii^	3.0500	H32F···C24A	2.9100
C65B···H12A^ii^	3.0200	H51A···C31A	2.9400
C66A···H51A	2.7900	H51A···C66A	2.7900
C66B···H51B	2.8000	H51A···H31A	2.3900
H1A···O4B	2.279 (16)	H51A···H66A	2.3500
H1A···C4B	3.073 (16)	H51A···O2B^ii^	2.8300
H1A···C22A	2.816 (14)	H51A···C12B^ii^	3.0100
H1B···C22B	2.823 (14)	H51A···H12D^ii^	2.4200
H1B···H32C^x^	2.3200	H51B···C66B	2.8000
H1B···O4A^x^	2.639 (16)	H51B···H31F	2.3200
H1B···C32A^x^	3.092 (16)	H51B···H66B	2.3000
H2A···H6A	2.2800	H51B···C31B	2.8800
H2A···H26A	2.3700	H52B···C63A	2.9600
H2A···H32B	2.4700	H52B···C62A	2.9900
H2B···H6B	2.3000	H62A···H6A	2.3100
H2B···H32E	2.4800	H62B···H6B	2.3100
H2B···H26B	2.4000	H63A···C16A	2.5200
H6A···H2A	2.2800	H63A···H16B	2.2900
H6A···C31A^vii^	3.0000	H63A···H16C	2.3200
H6A···H62A	2.3100	H63B···H16E	2.1500
H6A···H22A^vii^	2.4300	H63B···H16F	2.4600
H6A···H31A^vii^	2.5200	H63B···C16B	2.5100
H6B···C31B^vii^	3.0000	H63B···H32A^xii^	2.3900
H6B···H2B	2.3000	H65B···O2A^ii^	2.9100
H6B···H31F^vii^	2.4500	H66A···H51A	2.3500
H6B···H62B	2.3100	H66A···C5A	2.9300
H12A···O6A^iii^	2.6000	H66B···C5B	2.8800
H12A···C65B^iii^	3.0200	H66B···H51B	2.3000
			
C12A—O2A—C24A	116.51 (13)	C62A—C63A—H63A	120.00
C16A—O6A—C64A	117.66 (13)	C64A—C65A—H65A	120.00
C12B—O2B—C24B	116.02 (14)	C66A—C65A—H65A	120.00
C16B—O6B—C64B	116.55 (13)	C61A—C66A—H66A	119.00
C2A—N1A—C6A	111.88 (12)	C65A—C66A—H66A	119.00
C6A—N1A—H1A	108.2 (8)	N1B—C2B—C21B	110.14 (11)
C2A—N1A—H1A	107.3 (10)	N1B—C2B—C3B	109.63 (12)
C2B—N1B—C6B	112.39 (12)	C3B—C2B—C21B	113.75 (11)
C6B—N1B—H1B	106.6 (9)	C2B—C3B—C31B	111.57 (11)
C2B—N1B—H1B	109.3 (11)	C2B—C3B—C4B	107.07 (11)
N1A—C2A—C3A	109.70 (13)	C4B—C3B—C32B	110.73 (12)
N1A—C2A—C21A	109.51 (11)	C31B—C3B—C32B	109.73 (12)
C3A—C2A—C21A	115.09 (11)	C4B—C3B—C31B	107.29 (13)
C4A—C3A—C32A	110.15 (12)	C2B—C3B—C32B	110.39 (13)
C31A—C3A—C32A	109.70 (12)	O4B—C4B—C5B	121.12 (16)
C4A—C3A—C31A	107.09 (14)	O4B—C4B—C3B	121.87 (15)
C2A—C3A—C31A	111.69 (11)	C3B—C4B—C5B	117.01 (14)
C2A—C3A—C32A	110.14 (13)	C4B—C5B—C6B	110.78 (14)
C2A—C3A—C4A	108.01 (11)	N1B—C6B—C5B	108.00 (14)
O4A—C4A—C3A	122.08 (15)	C5B—C6B—C61B	114.27 (14)
O4A—C4A—C5A	121.25 (16)	N1B—C6B—C61B	109.61 (13)
C3A—C4A—C5A	116.67 (13)	C2B—C21B—C26B	120.82 (13)
C4A—C5A—C6A	112.21 (14)	C2B—C21B—C22B	122.09 (14)
C5A—C6A—C61A	113.75 (13)	C22B—C21B—C26B	117.09 (14)
N1A—C6A—C61A	110.25 (13)	C21B—C22B—C23B	122.18 (15)
N1A—C6A—C5A	108.34 (14)	C22B—C23B—C24B	119.38 (16)
C2A—C21A—C26A	120.51 (12)	C23B—C24B—C25B	119.79 (16)
C22A—C21A—C26A	117.00 (14)	O2B—C24B—C25B	115.34 (16)
C2A—C21A—C22A	122.43 (14)	O2B—C24B—C23B	124.88 (16)
C21A—C22A—C23A	122.19 (15)	C24B—C25B—C26B	119.60 (17)
C22A—C23A—C24A	119.45 (13)	C21B—C26B—C25B	121.94 (16)
O2A—C24A—C25A	115.61 (14)	C62B—C61B—C66B	117.21 (15)
C23A—C24A—C25A	119.67 (15)	C6B—C61B—C62B	120.43 (15)
O2A—C24A—C23A	124.72 (14)	C6B—C61B—C66B	122.20 (15)
C24A—C25A—C26A	120.08 (16)	C61B—C62B—C63B	122.24 (16)
C21A—C26A—C25A	121.61 (15)	C62B—C63B—C64B	119.25 (16)
C62A—C61A—C66A	117.66 (15)	O6B—C64B—C65B	116.23 (15)
C6A—C61A—C66A	121.18 (15)	O6B—C64B—C63B	124.14 (15)
C6A—C61A—C62A	121.13 (15)	C63B—C64B—C65B	119.63 (15)
C61A—C62A—C63A	121.89 (16)	C64B—C65B—C66B	120.24 (16)
C62A—C63A—C64A	119.17 (16)	C61B—C66B—C65B	121.40 (16)
C63A—C64A—C65A	119.69 (15)	N1B—C2B—H2B	108.00
O6A—C64A—C65A	115.67 (15)	C3B—C2B—H2B	108.00
O6A—C64A—C63A	124.65 (15)	C21B—C2B—H2B	108.00
C64A—C65A—C66A	120.48 (17)	C4B—C5B—H51B	110.00
C61A—C66A—C65A	121.12 (16)	C4B—C5B—H52B	109.00
C3A—C2A—H2A	107.00	C6B—C5B—H51B	109.00
C21A—C2A—H2A	107.00	C6B—C5B—H52B	109.00
N1A—C2A—H2A	107.00	H51B—C5B—H52B	108.00
H51A—C5A—H52A	108.00	N1B—C6B—H6B	108.00
C4A—C5A—H51A	109.00	C5B—C6B—H6B	108.00
C4A—C5A—H52A	109.00	C61B—C6B—H6B	108.00
C6A—C5A—H52A	109.00	O2B—C12B—H12D	109.00
C6A—C5A—H51A	109.00	O2B—C12B—H12E	109.00
C5A—C6A—H6A	108.00	O2B—C12B—H12F	109.00
C61A—C6A—H6A	108.00	H12D—C12B—H12E	109.00
N1A—C6A—H6A	108.00	H12D—C12B—H12F	109.00
H12B—C12A—H12C	109.00	H12E—C12B—H12F	109.00
O2A—C12A—H12A	109.00	O6B—C16B—H16D	109.00
H12A—C12A—H12B	109.00	O6B—C16B—H16E	109.00
H12A—C12A—H12C	109.00	O6B—C16B—H16F	109.00
O2A—C12A—H12B	109.00	H16D—C16B—H16E	109.00
O2A—C12A—H12C	109.00	H16D—C16B—H16F	109.00
O6A—C16A—H16A	109.00	H16E—C16B—H16F	109.00
H16B—C16A—H16C	109.00	C21B—C22B—H22B	119.00
O6A—C16A—H16B	109.00	C23B—C22B—H22B	119.00
O6A—C16A—H16C	109.00	C22B—C23B—H23B	120.00
H16A—C16A—H16B	109.00	C24B—C23B—H23B	120.00
H16A—C16A—H16C	109.00	C24B—C25B—H25B	120.00
C21A—C22A—H22A	119.00	C26B—C25B—H25B	120.00
C23A—C22A—H22A	119.00	C21B—C26B—H26B	119.00
C24A—C23A—H23A	120.00	C25B—C26B—H26B	119.00
C22A—C23A—H23A	120.00	C3B—C31B—H31D	109.00
C26A—C25A—H25A	120.00	C3B—C31B—H31E	109.00
C24A—C25A—H25A	120.00	C3B—C31B—H31F	109.00
C21A—C26A—H26A	119.00	H31D—C31B—H31E	109.00
C25A—C26A—H26A	119.00	H31D—C31B—H31F	109.00
C3A—C31A—H31C	109.00	H31E—C31B—H31F	109.00
H31A—C31A—H31B	109.00	C3B—C32B—H32D	109.00
C3A—C31A—H31B	109.00	C3B—C32B—H32E	109.00
C3A—C31A—H31A	109.00	C3B—C32B—H32F	109.00
H31A—C31A—H31C	109.00	H32D—C32B—H32E	109.00
H31B—C31A—H31C	109.00	H32D—C32B—H32F	109.00
H32A—C32A—H32B	109.00	H32E—C32B—H32F	109.00
C3A—C32A—H32B	109.00	C61B—C62B—H62B	119.00
H32A—C32A—H32C	109.00	C63B—C62B—H62B	119.00
C3A—C32A—H32C	109.00	C62B—C63B—H63B	120.00
C3A—C32A—H32A	109.00	C64B—C63B—H63B	120.00
H32B—C32A—H32C	109.00	C64B—C65B—H65B	120.00
C63A—C62A—H62A	119.00	C66B—C65B—H65B	120.00
C61A—C62A—H62A	119.00	C61B—C66B—H66B	119.00
C64A—C63A—H63A	120.00	C65B—C66B—H66B	119.00
			
C12A—O2A—C24A—C23A	12.1 (2)	C62A—C61A—C66A—C65A	0.2 (3)
C12A—O2A—C24A—C25A	−168.43 (13)	C66A—C61A—C62A—C63A	−0.1 (3)
C16A—O6A—C64A—C63A	1.5 (2)	C61A—C62A—C63A—C64A	0.0 (3)
C16A—O6A—C64A—C65A	−178.59 (15)	C62A—C63A—C64A—C65A	0.1 (3)
C12B—O2B—C24B—C25B	−169.55 (15)	C62A—C63A—C64A—O6A	−179.98 (14)
C12B—O2B—C24B—C23B	10.2 (2)	C63A—C64A—C65A—C66A	−0.1 (3)
C16B—O6B—C64B—C63B	12.0 (2)	O6A—C64A—C65A—C66A	−180.00 (17)
C16B—O6B—C64B—C65B	−168.29 (14)	C64A—C65A—C66A—C61A	−0.1 (3)
C6A—N1A—C2A—C3A	66.28 (14)	C21B—C2B—C3B—C31B	−60.64 (17)
C2A—N1A—C6A—C5A	−63.66 (16)	N1B—C2B—C3B—C32B	−174.56 (11)
C2A—N1A—C6A—C61A	171.27 (13)	C21B—C2B—C3B—C4B	−177.75 (13)
C6A—N1A—C2A—C21A	−166.52 (12)	N1B—C2B—C21B—C26B	145.00 (14)
C2B—N1B—C6B—C5B	−63.96 (16)	C3B—C2B—C21B—C22B	89.25 (17)
C6B—N1B—C2B—C21B	−168.54 (12)	C3B—C2B—C21B—C26B	−91.48 (16)
C2B—N1B—C6B—C61B	170.98 (12)	N1B—C2B—C3B—C31B	63.15 (15)
C6B—N1B—C2B—C3B	65.58 (14)	C21B—C2B—C3B—C32B	61.65 (15)
C3A—C2A—C21A—C22A	85.52 (16)	N1B—C2B—C21B—C22B	−34.27 (17)
C3A—C2A—C21A—C26A	−97.35 (15)	N1B—C2B—C3B—C4B	−53.96 (14)
N1A—C2A—C21A—C26A	138.54 (13)	C2B—C3B—C4B—C5B	49.08 (18)
N1A—C2A—C21A—C22A	−38.59 (17)	C32B—C3B—C4B—O4B	−10.1 (2)
N1A—C2A—C3A—C4A	−54.13 (14)	C32B—C3B—C4B—C5B	169.46 (14)
C21A—C2A—C3A—C32A	61.54 (15)	C2B—C3B—C4B—O4B	−130.49 (16)
N1A—C2A—C3A—C31A	63.39 (15)	C31B—C3B—C4B—O4B	109.62 (18)
C21A—C2A—C3A—C31A	−60.62 (17)	C31B—C3B—C4B—C5B	−70.82 (17)
C21A—C2A—C3A—C4A	−178.14 (12)	C3B—C4B—C5B—C6B	−50.2 (2)
N1A—C2A—C3A—C32A	−174.46 (11)	O4B—C4B—C5B—C6B	129.34 (17)
C32A—C3A—C4A—C5A	166.77 (14)	C4B—C5B—C6B—C61B	175.78 (15)
C2A—C3A—C4A—O4A	−133.38 (16)	C4B—C5B—C6B—N1B	53.53 (18)
C32A—C3A—C4A—O4A	−13.1 (2)	N1B—C6B—C61B—C62B	−106.34 (18)
C2A—C3A—C4A—C5A	46.45 (18)	C5B—C6B—C61B—C66B	−52.6 (2)
C31A—C3A—C4A—C5A	−73.99 (17)	C5B—C6B—C61B—C62B	132.31 (17)
C31A—C3A—C4A—O4A	106.18 (18)	N1B—C6B—C61B—C66B	68.76 (19)
O4A—C4A—C5A—C6A	133.11 (17)	C22B—C21B—C26B—C25B	−0.4 (2)
C3A—C4A—C5A—C6A	−46.7 (2)	C2B—C21B—C26B—C25B	−179.75 (14)
C4A—C5A—C6A—C61A	174.80 (15)	C2B—C21B—C22B—C23B	178.75 (14)
C4A—C5A—C6A—N1A	51.82 (18)	C26B—C21B—C22B—C23B	−0.6 (2)
C5A—C6A—C61A—C66A	−57.7 (2)	C21B—C22B—C23B—C24B	0.3 (3)
N1A—C6A—C61A—C62A	−113.79 (17)	C22B—C23B—C24B—C25B	0.9 (3)
N1A—C6A—C61A—C66A	64.2 (2)	C22B—C23B—C24B—O2B	−178.81 (15)
C5A—C6A—C61A—C62A	124.29 (17)	O2B—C24B—C25B—C26B	177.89 (15)
C26A—C21A—C22A—C23A	0.2 (2)	C23B—C24B—C25B—C26B	−1.9 (3)
C2A—C21A—C22A—C23A	177.45 (13)	C24B—C25B—C26B—C21B	1.6 (3)
C2A—C21A—C26A—C25A	−177.36 (14)	C6B—C61B—C66B—C65B	−174.47 (15)
C22A—C21A—C26A—C25A	−0.1 (2)	C62B—C61B—C66B—C65B	0.8 (2)
C21A—C22A—C23A—C24A	−0.7 (2)	C6B—C61B—C62B—C63B	174.43 (16)
C22A—C23A—C24A—C25A	1.0 (2)	C66B—C61B—C62B—C63B	−0.9 (2)
C22A—C23A—C24A—O2A	−179.58 (13)	C61B—C62B—C63B—C64B	−0.5 (3)
C23A—C24A—C25A—C26A	−0.9 (2)	C62B—C63B—C64B—C65B	2.0 (3)
O2A—C24A—C25A—C26A	179.66 (14)	C62B—C63B—C64B—O6B	−178.38 (15)
C24A—C25A—C26A—C21A	0.4 (2)	O6B—C64B—C65B—C66B	178.22 (15)
C6A—C61A—C66A—C65A	−177.91 (16)	C63B—C64B—C65B—C66B	−2.1 (3)
C6A—C61A—C62A—C63A	177.96 (15)	C64B—C65B—C66B—C61B	0.7 (3)

Symmetry codes: (i) −*x*+2, *y*+1/2, −*z*+1/2; (ii) −*x*+1, *y*−1/2, −*z*+1/2; (iii) −*x*+1, *y*+1/2, −*z*+1/2; (iv) −*x*+1, −*y*+1, −*z*+1; (v) *x*+1, −*y*+1/2, *z*−1/2; (vi) *x*, −*y*+1/2, *z*−1/2; (vii) *x*+1, *y*, *z*; (viii) −*x*+2, *y*−1/2, −*z*+1/2; (ix) −*x*+2, −*y*, −*z*+1; (x) *x*, −*y*+1/2, *z*+1/2; (xi) −*x*, −*y*+1, −*z*+1; (xii) *x*+1, −*y*+1/2, *z*+1/2; (xiii) *x*−1, −*y*+1/2, *z*−1/2; (xiv) *x*−1, *y*, *z*; (xv) *x*−1, −*y*+1/2, *z*+1/2.

### Hydrogen-bond geometry (Å, °)

**Table:**

*D*—H···*A*	*D*—H	H···*A*	*D*···*A*	*D*—H···*A*
N1A—H1A···O4B	0.92 (2)	2.28 (2)	3.1958 (17)	173.2 (14)
C25B—H25B···Cg1^i^	0.95	2.95	3.6993 (19)	137
C32A—H32B···Cg2^vi^	0.98	2.82	3.4573 (19)	124
C5B—H52B···Cg1	0.99	2.97	3.7989 (19)	142

Symmetry codes: (i) −*x*+2, *y*+1/2, −*z*+1/2; (vi) *x*, −*y*+1/2, *z*−1/2.

## References

[bb1] Dimmock, J. R., Arora, V. K., Wonko, S. L., Hamon, N. W., Quail, J. W., Jia, Z., Warrington, R. C., Fang, W. D. & Lee, J. S. (1990). *Drug Des. Deliv.* **6**, 183–194. 2076179

[bb2] Farrugia, L. J. (1997). *J. Appl. Cryst.* **30**, 565.

[bb3] Gayathri, P., Thiruvalluvar, A., Manimekalai, A., Sivakumar, S. & Butcher, R. J. (2008). *Acta Cryst.* E**64**, o1973. 10.1107/S1600536808029723 PMC2959467 21201173

[bb4] Mutus, B., Wagner, J. D., Talpas, C. J., Dimmock, J. R., Phillips, O. A. & Reid, R. S. (1989). *Anal. Biochem.* pp. 237–243. 10.1016/0003-2697(89)90045-6 2729541

[bb5] Oxford Diffraction (2008). *CrysAlis CCD* and *CrysAlis RED* Oxford Diffraction Ltd, Abingdon, Oxfordshire, England.

[bb6] Sheldrick, G. M. (2008). *Acta Cryst.* A**64**, 112–122. 10.1107/S0108767307043930 18156677

[bb7] Spek, A. L. (2003). *J. Appl. Cryst.* **36**, 7–13.

